# Development of a Univariate Membrane-Based Mid-Infrared Method for Protein Quantitation and Total Lipid Content Analysis of Biological Samples

**DOI:** 10.1155/2014/657079

**Published:** 2014-10-13

**Authors:** Ivona Strug, Christopher Utzat, Amedeo Cappione, Sara Gutierrez, Ryan Amara, Joseph Lento, Florian Capito, Romas Skudas, Elena Chernokalskaya, Timothy Nadler

**Affiliations:** ^1^EMD Millipore Corporation, 17 Cherry Hill Drive, Danvers, MA 01923, USA; ^2^Institute for Organic Chemistry and Biochemistry, Technical University Darmstadt, 64289 Darmstadt, Germany; ^3^Merck KGaA, Frankfurter Straße 250, 64293 Darmstadt, Germany

## Abstract

Biological samples present a range of complexities from homogeneous purified protein to multicomponent mixtures. Accurate qualification of such samples is paramount to downstream applications. We describe the development of an MIR spectroscopy-based analytical method offering simultaneous protein quantitation (0.25–5 mg/mL) and analysis of total lipid or detergent species, as well as the identification of other biomolecules present in biological samples. The method utilizes a hydrophilic PTFE membrane engineered for presentation of aqueous samples in a dried format compatible with fast infrared analysis. Unlike classical quantification techniques, the reported method is amino acid sequence independent and thus applicable to complex samples of unknown composition. By comparison to existing platforms, this MIR-based method enables direct quantification using minimal sample volume (2 *µ*L); it is well-suited where repeat access and limited sample size are critical parameters. Further, accurate results can be derived without specialized training or knowledge of IR spectroscopy. Overall, the simplified application and analysis system provides a more cost-effective alternative to high-throughput IR systems for research laboratories with minimal throughput demands. In summary, the MIR-based system provides a viable alternative to current protein quantitation methods; it also uniquely offers simultaneous qualification of other components, notably lipids and detergents.

## 1. Introduction

Correct estimation of protein concentration in aqueous biological samples is an essential step in biochemical research and the pharmaceutical industry impacting downstream applications ranging from biomarker studies to quality control in the production of biotherapeutics. Determination of protein concentration in most popular assays is accomplished via comparison to a sequence-based extinction coefficient (UV measurements) or in relation to a standard (traditional dye-based absorbance assays such as BCA, Lowry, and Bradford) [1–3]. However, most recent reviews point out the fact that, due to assay-specific limitations, there is no single method dominating protein quantification [1, 3]. While UV based quantification is reliant upon absorbance of tryptophan, tyrosine, and cysteine at 280 nm [4, 5], a protein's extinction coefficient can vary widely with sequence. In fact, a greater than two-fold difference is observed between extinction coefficients calculated for albumin and immunoglobulin G. Also, mixtures of unknown composition (most notably, biologically relevant samples) cannot be confidently quantified based on absorption at 280 nm. Colorimetric assays are strongly influenced by the presence of detergents and other reagents. Moreover, amino acid analysis (AAA) is capable of delivering possibly the most accurate protein quantitation [2], but the method is expensive with lengthy turnaround times if samples are sent to a third party for analysis. Performed in-house, AAA requires time-consuming sample manipulation and specialized equipment.

Infrared (IR) spectroscopy is a powerful and growing analytical tool for the detection and analysis of biomedically relevant compounds such as proteins, lipids, carbohydrates, and nucleic acids [6–9]. Midinfrared (MIR) spectroscopy is based on the absorption of radiation in the approximate range 4000–400 cm^−1^ and is currently considered among the most promising spectroscopic techniques for application in biomedical research and diagnostics [10–12]. Also, MIR spectroscopy has been recognized as a viable method for lipid analysis [13–16] and is one of the oldest and well established experimental techniques for the analysis of protein and carbohydrate structure [17–21]. Attenuated total reflection (ATR) spectroscopy and transmission flow-through cells used in combination with complex chemometric data analysis have recently enabled fast quantitative protein analysis directly from aqueous samples [22–29]. However, while flow-through cells for protein quantification allow for automated sample analysis, these devices have a propensity for clogging, requiring frequent, time-consuming cleaning procedures. While ATR cells are more robust, the required sample volumes (10–25 *μ*L) may be considered significant, particularly in situations where biological samples, limited by both volume and repeat access, are to be analyzed. Much less sample is required for ATR-based measurements performed on dried samples. However, due to enhanced sensitivity, the multivariate approach (e.g., partial least-squares analysis (PLS)) is usually applied to data analysis [14, 22–29] reducing attractiveness of the method for routine application in biological laboratories that usually lack the time and expertise required for method development and validation. To our knowledge, an easy, fast, and robust method utilizing ATR and univariate data analysis for accurate and reproducible protein quantitation from complex biological samples in a dried format has not yet been reported.

Several Amide bands have been identified in MIR spectroscopy allowing for characterization and quantification of proteins. Among these, Amide I (1600–1690 cm^−1^) and Amide II (1480–1575 cm^−1^) are recognized as the most representative of all vibration modes [17, 18]. The Amide I absorption consists predominantly of C=O stretching vibration (about 80%) with a minor contribution from the C–N stretching vibration (20%), while the Amide II band is more equally split between N–H bending (60%) and C–N stretching (40%) [30]. Until recently, analysis of the Amide I and II absorption regions has been severely hampered in aqueous samples due to spectral interference of a strong water absorption band at 1500–1700 cm^−1^ [25]. While the advent of ATR and flow-through cells has circumvented the water interference issues, their utility is limited due to practical drawbacks which include instrumentation cost, expertise, and time required for method development and accurate data analysis.

A simple univariate (Beer-Lambert) analysis, applied in the method reported here, relies on the integration of Amide I band and uses directly searchable absorptions on the spectrum curve. Reported protein quantification by MIR, while still based on a curve-fitting technique, presents substantial advantages over other current methodologies like UV absorbance or colorimetric assays. First, unlike UV absorbance at 280 nm, MIR-based protein quantitation is much less dependent upon amino acid composition. Also, Amide bond quantitation by MIR is not subject to signal interference from many common biological buffer components such as detergents, reducing agents, and chelators, demonstrating superiority over standard colorimetric assays. Moreover, the MIR-based method enables fast and accurate peptide quantitation providing researchers with a robust substitute for time-consuming amino acid analysis. However, when compared to UV spectroscopy, IR instruments are more costly and require technical expertise as well as time-consuming method development preventing widespread applicability of MIR for general protein quantification. Thus, an instrument or method, allowing for simple and more cost-effective analysis of samples, while at the same time combining the advantages of ATR and flow-through based systems, would be of unique value. Additionally, in contrast to UV or any other known protein quantitation method, simple, MIR-based analysis can also be employed for simultaneous analysis of lipids or detergents [14, 30–36]. Due to their complex and varied chemical composition, lipids absorb in many different regions of the IR spectrum. Characteristic lipid bands, such as the aliphatic group stretching (3000–2800 cm^−1^), ester C=O stretching (around 1740 cm^−1^), or phosphate stretching (around 1235 cm^−1^) permit qualitative and quantitative analysis of lipid content [14, 31, 32]. Due to similarities in composition, detergents possess MIR absorption spectra that closely resemble lipids present in the cell membrane and can be analyzed along with lipids. This commonality offers researchers a means for monitoring the efficiency of residual detergent removal from lysate-derived samples prior to downstream application.

The method reported in this paper uses a hydrophilic polytetrafluoroethylene (PTFE) membrane engineered for sample application and retention. The membrane is transparent in the MIR regions used for protein and lipid/detergent analysis. The size of the sample application spot was further designed to be slightly smaller than the IR beam, ensuring probing of the entire sample. This constraining feature is important in enabling simple, univariate, quantitative measurements. In comparison to currently available techniques and instruments (e.g., HTS-XT system by Bruker Optics), the method described here is technically less complex; thus, it is more cost-effective and especially well suited for routine analysis of small sample numbers. Also, given the minimal volume (2 *μ*L) required for measurement, this method can be successfully applied for the analysis of precious material available in limited quantities.

The presented paper provides a detailed description of method development along with a comparison to other protein quantification techniques routinely used in biological laboratories, with respect to the required sample volume, time-consumption, labor-intensity, accuracy, and robustness. In addition to general protein quantification, the ability for simultaneous lipid analysis was also investigated. Well characterized solutions of several purified proteins, protein mixtures, and examples of lipids and detergents were used to assess quantification limits, dynamic range, linearity, accuracy, precision, and robustness of the reported method. Further applicability of the method for the analysis of biological samples was demonstrated using cell lysates and tissue homogenates.

## 2. Materials and Methods

### 2.1. Sample Carrier Design

**Figure 1:**
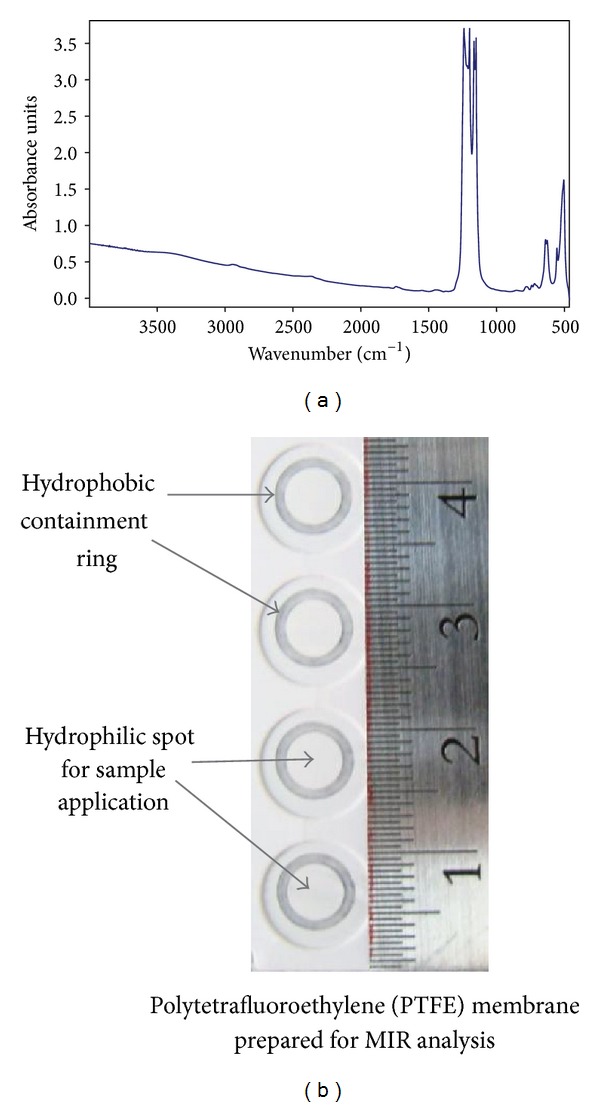
Development of a disposable sample carrier and optimization of “spot” design; (a) MIR signature of PTFE membrane; (b) the design of sample card allowing for containment of analyzed samples within the MIR beam.

In order to avoid cleaning steps in between series of successive measurements, a disposable device for sample application was designed. From several investigated options, a membrane-based system emerged as the most cost-efficient and the easiest to use, predominantly because it allowed rapid water removal and drying of the sample. Various membranes were tested (data not shown); ultimately a hydrophilic PTFE-based membrane was selected due to its transparency within the relevant MIR regions, allowing for the quantification of proteins and lipids. As shown in Figure 1(a), the membrane spectrum contains a strong signal between 1100 and 1300 cm^−1^ but is transparent in the MIR region above 1300 cm^−1^ used for the analysis of biological samples reported here. The hydrophilic PTFE membrane (30 *μ*m thick) is mounted on cardboard frame to allow easier handling and provide a place to record sample names, while also assuring consistent presentation for interrogation by the MIR beam. The card configuration contains four analysis areas designed for easy application and containment of the entire sample within the focused IR beam. The hydrophilic spot for sample application (4.5 mm diameter; see Figure 1(b) and Supplementary Figure S1A, the Supplementary Material will be available online at http://dx.doi.org/10.1155/2014/657079.) is surrounded by a hydrophobic ring generated by mechanical removal of hydrophilic surface; the ring prevents analyte dispersal promoting precise presentation of the entire sample to the MIR beam (diameter ≥ 6.5 mm) [37, 38]. Samples are spotted directly to the membrane without any additional preparation steps. The reported method permits two ways of drying aqueous sample spotted onto PTFE membrane. On average, 2 *μ*L of sample placed on the membrane spot requires around 10 to 15 minutes to dry completely at room temperature. The sample can also be dried within around 30 seconds, on average, by the heater and fan located in the sample carrier chamber of the dedicated FTIR spectrometer. A “4-spot” card was selected for the final configuration to allow rapid analysis of an individual sample in triplicate or three separate samples in comparison to an appropriate background buffer “spot.”

### 2.2. Data Collection and Analysis

Measurements were performed using the Direct Detect spectrometer (EMD Millipore), a Fourier transform (FT)-IR system analyzing the spectral range 500–6000 cm^−1^. All spectra were derived from dried samples in transmission mode. A classical univariate quantification method, based on the fact that MIR spectroscopy of nonscattering samples obeys Beer's law, was applied to data analysis. For each protein and lipid/detergent measurement, an appropriate background (buffer) spectrum was collected. A buffer subtraction step was performed only on regions of the spectra used for Amide I and/or aliphatic symmetric stretching signal analysis. Proteins are quantified using the Amide I region (1702–1602 cm^−1^) only; thus, all other regions of the MIR spectrum are not critical for the analysis. Consequently, for accurate protein quantification, it was sufficient to subtract buffer in the region (1850–1350 cm^−1^) surrounding Amide I area. The same procedure was applied to aliphatic stretching region used for lipid/detergent analysis. Buffer subtraction is performed between 3100 and 2600 cm^−1^; the rest of MIR spectra is not being utilized. Buffer subtracted spectral regions are used for appropriate band integration.

Amide I band integration is performed by anchoring the baseline at fixed points between 1702 and 1602 cm^−1^ and determining the Amide I signal value at the highest point between these wavenumbers. Two additional integration methods (not discussed here) accounting for possible buffer interference have also been developed.

Aliphatic symmetric stretching band integration is performed by anchoring the baseline between 2990 and 2810 cm^−1^ and determining the signal strength at the highest point between 2868 and 2838 cm^−1^.

### 2.3. Database Interrogation

To compare the theoretical variability of various quantitation methods, a protein database was examined. For example, the theoretical extinction coefficient at 280 nm is based on the number of tryptophan, tyrosine, and cysteine residues in the protein [5]. The MIR-based analysis is dependent on the number of Amide bonds, which not only link the amino acids together but are also present in the side chains of asparagine and glutamine. To automate the examination of the database, a Visual Basic for Applications (VBA) program was written for Excel (Microsoft) to check all 20233 proteins in the human protein database (Uniprot Release 2012_10, [39]) for (1) total count of amino acids, (2) number of glutamines, (3) number of asparagines, (4) number of tryptophans, (5) total molecular weight, and (6) theoretical molar extinction coefficient at 280 nm. The theoretical molar extinction coefficient was estimated by summing the number of tryptophans in the protein multiplied by 5690, the number of tyrosines by 1280, and the number of cysteines by 120. This molar extinction coefficient was converted to a mass coefficient by dividing by the molecular weight of the protein to derive an extinction coefficient with units of AU mL mg^−1^ cm^−1^. The average mass per residue was estimated by dividing the molecular weight by the number of amino acids in the protein. The number of Amides was determined by adding the number of asparagines and glutamines to the total number of amino acids and subtracting one. The average mass per Amide was determined by dividing the molecular weight by the number of Amides. Note that posttranslational modifications were not taken into account.

### 2.4. Protein Quantitation

Sample protein concentrations were determined with reference to a calibration curve; the method requires prior generation of a reusable standard curve derived from serial dilution of a reference protein. For all reported experiments, the system was calibrated using bovine serum albumin (BSA) from the National Institute of Standards and Technology (NIST) diluted in phosphate-buffered saline (PBS). A series of ten concentration points (0.125–5 mg/mL) was used to generate the protein calibration curve. All reported measurements were performed in triplicate using 2 *μ*L of sample solution per membrane position. From pilot experiments (not discussed here), 2 *μ*L was chosen as a trade-off between minimum sample volume required and acceptable quantitation results. Although smaller quantities could be applied to the membrane, human error in the pipetting of such low volumes would introduce additional variability and thus was not considered further.

Performance of the method, within the standard curve-defined dynamic range, was assessed using pure protein solutions as well as protein mixtures. Pure protein solutions were prepared with lysozyme solubilized in Milli-Q water and protein A in PBS. Protein mixtures consisted of BSA, cytochrome C, alcohol dehydrogenase, human transferrin, concanavalin A, lysozyme, *ϒ*-globulins from rabbit, and protein A in PBS, used at two distinct concentrations.

For reference purposes, the concentration of all examined protein solutions was determined by amino acid analysis (AAA). To obtain a 1 mg/mL solution analyzed by MIR-based quantitation, a lysozyme sample (AAA determination at 68 mg/mL) was diluted with PBS at 1/68 ratio. Protein A (AAA determination at 52 mg/mL) was diluted, also with PBS, 1/13 to obtain 4 mg/mL sample. The protein mixture (AAA determination at 1.98 mg/mL) was used at the AAA estimated concentration as well as a 1/8 dilution.

Potential interference from detergents and reducing agents, which are known to impact Bradford and Lowry protein determination assays, was investigated using known concentrations of BSA in PBS (from 0.25 to 2 mg/mL) spiked with increasing amount of investigated additive. Influence of Tween 20 and Triton X-100 on protein quantitation was tested in the range of 0.1–5% detergent. *β*-mercaptoethanol (BME) and DL-dithiothreitol (DTT) were analyzed at up to 150 mM and 50 mM, respectively. Possible obstruction of protein quantification due to the presence of sodium dodecyl sulfate (SDS) was analyzed using buffers containing up to 1% of the detergent. While all investigated detergents and reducing agents produced MIR spectra, none was absorbing within the region used for protein quantification (1700–1600 cm^−1^).

### 2.5. Lipid and Detergent Analysis

Empirical sample concentration values were determined by interpolation from calibration curves developed for each lipid or detergent analyzed. The reported method relied on strength of bands produced by vibrations of aliphatic groups (3000–2800 cm^−1^). For the experiments reported here, the system was calibrated using tetracosanoic acid in chloroform and 3-[(3-cholamidopropyl) dimethylammonio]-1-propanesulfonate (CHAPS) in PBS. A series of seven concentrations (0.25–1.75 mg/mL) was used to generate a calibration curve for tetracosanoic acid. For CHAPS, the calibration curve was also derived from seven concentration points (0.25–4%). Unknown lipid mixtures were analyzed based solely on the strength of MIR signal.

### 2.6. Single Step Protein and Lipid Analysis in Complex Biological Samples

To investigate whether protein and lipid can be quantified simultaneously in complex biological samples, tissue lysates, originating from breast cancer tissues, were analyzed. Surgical frozen tissue, derived from a human breast ductal carcinoma, was obtained from Analytical Biological Services Inc. and divided into 2 equal samples (115 mg each). Tissue was covered with 2 mL 1x RIPA buffer (EMD Millipore; final composition: 50 mM Tris-HCl pH 7.4, 150 mM NaCl, 0.25% deoxycholic acid, 1% NP-40, and 1 mM EDTA) or CytoBuster protein extraction reagent (EMD Millipore; composition not available), both supplemented with an inhibitor cocktail and disrupted with a glass tissue homogenizer. Liquid fractions from homogenized and lysed tissue were transferred to separate tubes (fraction 1, Supplementary Figure S2) while the remaining pieces of tissue were covered with fresh volume of corresponding lysis buffer and homogenized again (fraction 2, Supplementary Figure S2). All samples were centrifuged at 10,000 ×g for 10 minutes. The centrifugation resulted in small pellets and double-layered supernatants. Protein containing fractions (bottom of the supernatant layers (P1a and P2a)) were separated and analyzed, using MIR-based method, for protein and lipid content. The top layers were saved for future lipid analysis. To remove substantial amounts of lipids detected in both protein fractions, an additional centrifugation step (15,000 ×g for 10 minutes) was introduced. Resulting layers, top lipid fraction (L1b and L2b), and bottom protein fractions (P1b and P2b) were analyzed, using MIR-based method, for protein and lipid content. Protein fractions were further utilized in downstream immunodetection of breast cancer biomarkers.

Lysates were prepared from MCF-7 (ATCC HTB-22) breast cancer cells (1.4 × 10^6^ cells per sample) in 1 mL of either 1x RIPA buffer or CytoBuster protein extraction reagent, both supplemented with protease inhibitor cocktail, by homogenization for few seconds with a handheld homogenizer followed by centrifugation at 15,000 ×g for 10 minutes. The MIR-based method was used to measure both protein and lipid content of the supernatant. MIR-based protein data was compared to the results obtained using BCA protein assay kit (Thermo Scientific). The supernatant was further used in breast cancer biomarkers analysis.

## 3. Results

### 3.1. Sample Card Design for Accurate MIR-Based Analysis of Biological Samples

MIR-based analysis of biological samples was achieved by the application of 2 *μ*L aqueous samples onto hydrophilic PTFE membrane and the presentation of dried sample to the MIR beam. While ZnSe and calcium fluoride are commonly used in MIR spectroscopy, they are cost-prohibitive for single-use applications. Also, the intention of the presented method was to avoid cleaning of the deposition window after each use and PTFE-based single-use card permitted such application. The PTFE membrane displays strong signal between 1100 and 1300 cm^−1^ (Figure 1(a)); however, it is relatively inert in the remaining MIR spectrum, including the Amide I region used for protein quantitation and the aliphatic group stretching region used for lipid and detergent measurement.

During development, it became clear that precise overlap between the site of sample application and the MIR beam is critical for quantitative accuracy. In order to achieve highly reproducible sample presentation to the MIR beam, a hydrophobic ring was introduced through mechanical membrane crushing and removal of the hydrophilic surface. Introduction of this ring allowed for precise confinement of applied sample within the designated spot. For all future measurements, the membrane “spot” was surrounded by a hydrophobic embossment (Figure 1(b)) preventing dispersion of the aqueous sample during application and drying. The “spot” design significantly improved the overlap between the MIR beam intensity profile, being the strongest in the center of the spotting area, and the dried sample area, thereby promoting higher assay accuracy. Because the comparative measurements are performed on dried samples, and therefore volume-dependent, reproducible and precise deposition of the employed 2 *μ*L onto the membrane is critical. All results reported here were obtained using manual pipette (Rainin, Pipet-Plus R2) with a latch trigger mechanism and aspiration rate controller, features that improve precision from sample to sample and from operator to operator.

### 3.2. Protein Analysis

In order to enable a fast, cost-effective, and simple analysis method, univariate quantification was applied. Although multivariate quantification procedures can provide better sensitivity when applied to the analysis of complex samples, they require IR expertise. As the method described here is intended for general protein quantification that is rapid and straightforward to non-IR specialists, the simpler univariate approach was pursued.

The influence of protein secondary structure on Amide I extinction coefficient (exact location of Amide I band) has been well documented [17, 18, 40]. Different spectral regions within the Amide I area were analyzed and the best results for general protein quantification were obtained for the region between 1702 and 1602 cm^−1^. Additionally, to account for possible buffer interference, a buffer signal (e.g., originating from buffer salts deposited and dried on the membrane) was subtracted from the protein signal. Investigation of various spectral regions considered for buffer signal subtraction delivered the most promising results for the region between 1850 and 1350 cm^−1^. Following subtraction, the strength of the remaining Amide I signal was used to interpolate the estimated protein concentration from a known standard curve.

Under standard conditions, aqueous samples spotted on hydrophilic materials, including PTFE membrane, dry forming a “coffee ring” effect where the majority of sample is preferentially deposited around the edges of a spot [41]. Distribution of the “coffee ring” is strongly influenced by the nature of the sample buffer. The most pronounced “coffee ring” effect is displayed in water (Supplementary Figure S1B) while use of buffers, such as PBS, reduces water tension allowing more uniform sample distribution across the membrane surface (Supplementary Figure S1C). In the presence of detergent, the effect is minimized or completely eliminated (Supplementary Figure S1D). The buffer-dependent differences in dried sample distribution are highly reproducible but require generation of a reusable calibration curve and confirmation of linearity for each matrix to ensure the most accurate results.

#### 3.2.1. Considerations for the Choice of Protein Standard and Calibration Curve Generation

**Figure 2:**
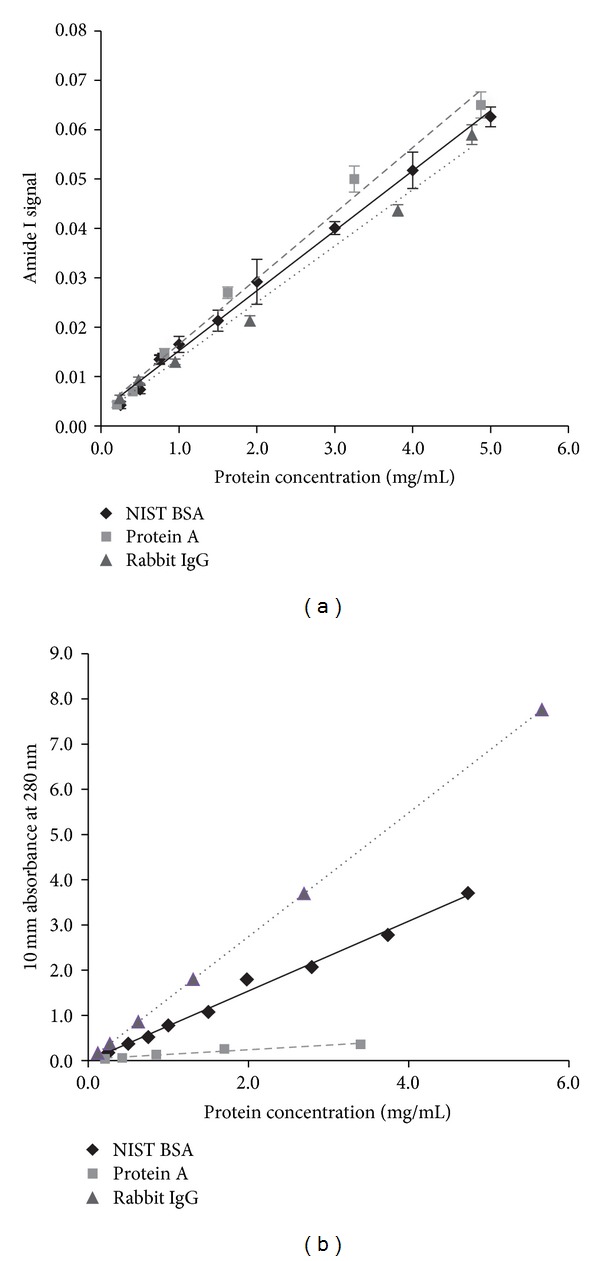
Comparison of quantification of three different proteins (BSA, protein A, and rabbit *ϒ*-globulins) using either the MIR-based approach (a) or UV_280_ spectroscopy (b). Unlike MIR, each protein curve determined by UV possessed a different slope displaying the influence of protein sequence content.

One major limitation of UV/Vis-based protein quantitation is its strict dependence on amino acid composition, in particular the presence of tryptophan and tyrosine. Comparative analysis between Amide I-derived signal and UV/Vis absorbance at 280 nm was performed for a range of concentrations of BSA, rabbit IgG, and protein A (Figure 2). Unlike UV/Vis, MIR-based analysis was unaffected by the proteins' vast differences in amino acid composition or size.

In reviewing the human protein database (Table 1), it is clear that many protein properties, such as protein length and mass, the number of specific amino acids, and even the predicted extinction coefficients at 280 nm, vary widely from protein to protein. However, the average mass per residue tends to be surprisingly consistent considering that individual residue contributions can range from 57 Da for glycine to 186 Da for tryptophan. Further, since the number of Amide bonds in a protein or peptide is only one less than the number of residues, plus the number of asparagines and glutamines, the mass per Amide bond is also very consistent, even for short proteins and peptides. One could therefore postulate that MIR absorbance (on a mass basis, not a molar basis) would be very consistent and that a single protein could serve as a reliable reference for just about any other protein or peptide, assuming that its average residue mass did not deviate significantly from the typical 110 Da per residue (e.g., polyglycine or polytryptophan would be expected to deviate).

**Figure 3:**
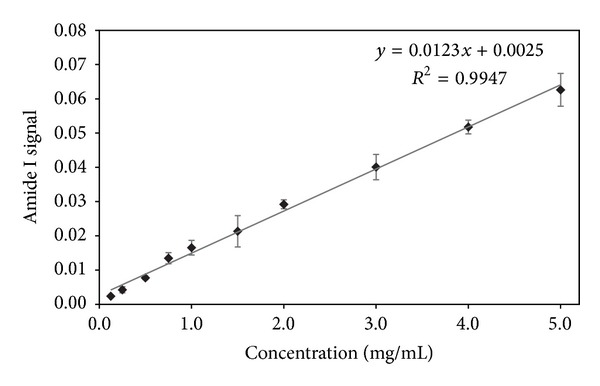
Protein calibration curve developed using NIST BSA diluted into PBS. A series of ten dilutions (in triplicate), spanning the range 0.125–5 mg/mL, was used to prepare a calibration curve. Amide I signal strength delivered by each concentration point was fitted to a regression line.

In order to test the MIR-based protein quantitation method, a FTIR spectrometer was calibrated using NIST BSA diluted into PBS. A series of ten dilutions (in triplicate), spanning the range 0.125–5 mg/mL, was used to prepare a calibration curve. Amide I signal strength delivered by each concentration point was fitted to a regression line that was ultimately used to determine protein concentration in the analyzed samples (Figure 3).

#### 3.2.2. Dynamic Concentration Range and Measurement Accuracy

Accuracy of MIR-based concentration estimation was determined using single-protein solutions as well as a protein mixture. Concentration values were derived by interpolation from a BSA reference curve and compared to amino acid analysis (AAA), a method recognized currently as the gold standard for estimating protein concentration. First, a 1 mg/mL lysozyme sample in PBS, prepared by 1/68 dilution of a 68 mg/mL sample (concentration determined by AAA) was found by the MIR method to be 0.922 ± 0.061 mg/mL. Next, protein A (52 mg/mL by AAA) was diluted 1/13 with PBS to obtain a 4 mg/mL solution. When analyzed by MIR, a concentration of 4.047 ± 0.184 mg/mL was determined. Lastly, a mixture of proteins (1.98 mg/mL by AAA) was quantified at 1.944 ± 0.028 mg/mL; a 1/8 dilution of the same sample (expected 0.25 mg/mL) delivered a concentration of 0.273 ± 0.028 mg/mL when analyzed by the MIR-based method.

Overall, the method showed very good accuracy and linearity in response to samples between 0.25 mg/mL and 5 mg/mL. Measurement accuracy for samples below 0.25 mg/mL decreased significantly; thus, a limit of 0.25 mg/mL was selected for this method. In regard to the upper detection limits, a small set of samples up to 100 mg/mL was successfully measured (data not shown). However, as the intended application of this method was for the analysis of precious samples, such high sample concentrations were not analyzed within the context of this paper.

#### 3.2.3. Reproducibility and Precision

Sample cards prepared using protein mixtures, at 0.25 and 1.98 mg/mL (each in triplicate; total of 9 spots per sample), were analyzed multiple times to determine measurement reproducibility. Each card (3 protein spots + 1 control spot) was measured four times and the concentrations obtained for each position as well as for an entire card were compared. Assuring correct and complete deposition of the samples onto the membrane, the average concentrations were 0.277 mg/mL (4.9% CV) and 1.942 mg/mL (1.5% CV), respectively. The precision at each individual card position was measured, with CVs of 1.3 and 0.3%, respectively, at position 2. Position 3 delivered data with CVs of 1.2 and 0.1%, while CVs at position 4 were at 2.3 and 0.1%, respectively. The greater precision found for the more concentrated sample is consistent with prior findings.

#### 3.2.4. Protein Quantitation in Buffers Containing Interfering Components, Detergents, and Reducing Agents

Protein quantitation method reported here relies on MIR-based evaluation of biological samples (from original buffers) spotted and dried onto a membrane. Therefore, measurements in the presence of buffer salts containing Amide bonds, such as urea, can potentially affect the accuracy of the results. In the majority of cases, interference from buffer salts is accounted for in the buffer subtraction step; however, since the method relies on a simple univariate approach, high concentrations of interfering components can still overwhelm the protein signal and preclude proper measurement.

**Figure 4:**
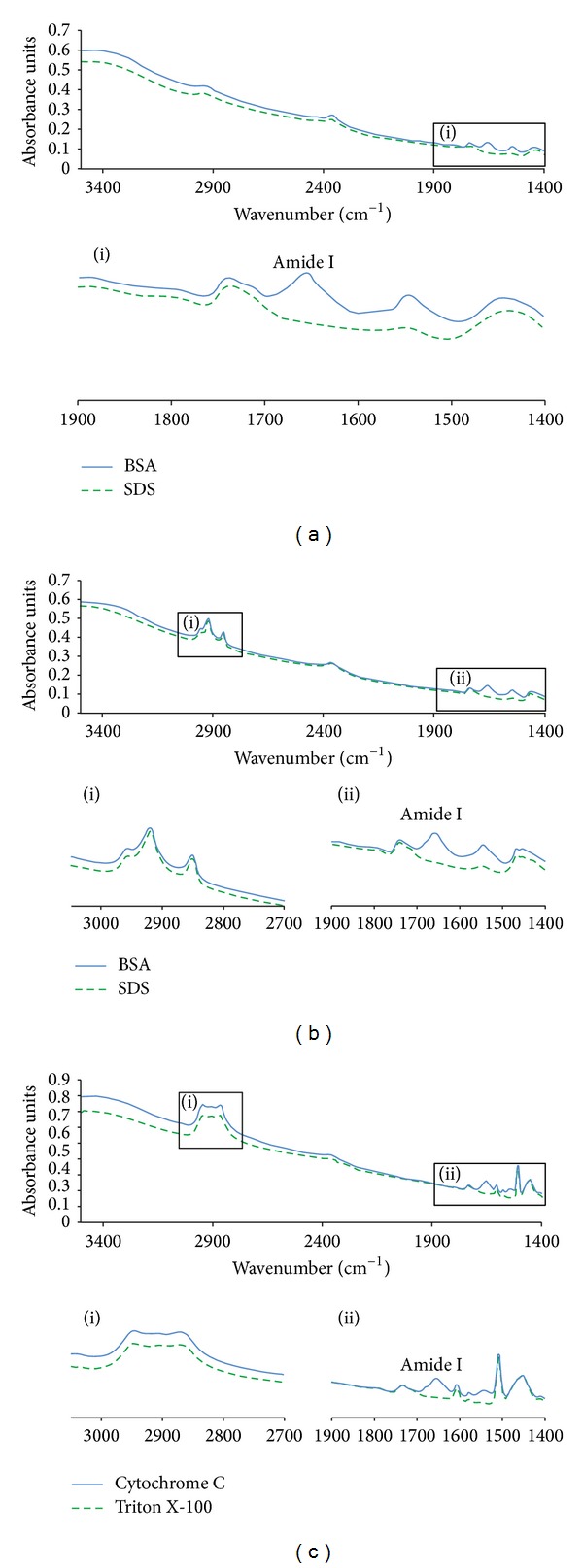
MIR-based protein quantitation in the presence of reducing agents and detergents. The top part of each box shows IR signal registered between 3500 and 1400 cm^−1^. The bottom part of each box shows a magnification of areas of the MIR spectrum characteristic of protein (1500–1700 cm^−1^) and detergent (2800–3000 cm^−1^) signals. The spectra of the buffers containing respective detergent are shown in green. The MIR spectra of protein in the detergent containing buffers are shown in blue. The samples analyzed are as follows: (a) 4 mg/mL BSA in the presence of DTT, (b) 4 mg/mL BSA in the presence of 1% SDS, and (c) 5 mg/mL cytochrome C in the presence of 5% Triton X-100.

Reducing agents and detergents are known to interfere with colorimetric protein quantitation methods [42]. To elucidate whether these agents would also interfere with MIR-based measurements, protein quantitation was performed in the presence of various additives. The results reveal that accurate MIR-based protein quantitation can be achieved in the presence of up to 50 mM dithiothreitol (DTT) (Figure 4(a)) and up to 150 mM of *β*-Mercaptoethanol (spectra not shown). In addition, the infrared absorption pattern of sodium dodecyl sulfate (SDS; analyzed up to 1%) did not overlap with the protein region allowing unbiased protein quantification in the presence of this detergent (Figure 4(b)). Method performance was also unaltered by the presence of up to 5% Tween 20 (data not shown) or Triton X-100 (Figure 4(c)). At the same time, the presence of Amide containing detergent, like, for example, CHAPS, might interfere with or prevent accurate protein quantitation. Also, since detergents are known to bind to proteins with varying affinities, subtracting the buffer contribution might not always be sufficient for accurate analysis. For example, in cases when analyzed proteins bind a significant amount of the detergent, the bound detergent will most likely not be accounted for by the blank influencing the accuracy of concentration determination.

### 3.3. Lipid and Detergent Analysis

#### 3.3.1. Calibration Curve Generation

By virtue of its ability to detect the spectral absorbance bands for many structural entities, the MIR-based method is not limited to the analysis of protein species. Given the large number of lipid-associated absorption bands, application of the described method for the quantification of lipid biomolecules was also investigated. Among lipid bands, the aliphatic C–H stretching region (2850–2870 cm^−1^) provides an ideal candidate for the analysis of lipids and detergents. While a single-protein standard can be used to quantify a wide range of protein and peptide samples, due to the vast complexity and variability among lipids and detergents, it was speculated that each quantitation would require the generation of a standard curve using the specific species in question. Experiments using various lipids, including fatty acids (Supplementary Figure S3A), phospholipids, triglycerides, liposaccharides, and many detergents, demonstrated a high degree of variability in the detection limits and slope of the employed calibration curves, thus confirming requirement for individualized calibrations for each of the analyzed lipids and detergents.

In order to validate the compatibility of MIR-based quantitation method for lipid and detergent analysis, the FTIR spectrometer was initially calibrated using either tetracosanoic acid (in chloroform) or CHAPS (in PBS). Two concentration ranges (performed in triplicate), spanning 0.25–1.75 mg/mL (tetracosanoic acid) and 0.25–4% (CHAPS), were used to derive lipid and detergent calibration curves (Supplementary Figure S3B). The strength of symmetric C–H vibration for each concentration was fitted to a regression line.

#### 3.3.2. Accuracy of Lipids and Detergent Quantification

The accuracy of concentration estimation within the established dynamic ranges for tetracosanoic acid and CHAPS was assessed using 0.8 mg/mL tetracosanoic acid and 1.8% CHAPS. The results showed that, for a well defined calibration range, the method was capable of estimating lipid and detergent concentration with low error. Assuring precise pipetting when applying the samples, MIR-based quantitation of the tetracosanoic acid sample returned 0.853 ± 0.14 mg/mL (2.4% CV) and CHAPS sample was quantified as 1.8 ± 0.004% (2.3% CV).

### 3.4. Single Step Protein and Lipid Analysis in Complex Biological Samples

The experiments using several formulations of lysis buffers, like RIPA and CytoBuster protein extraction reagent, spiked with known concentration of BSA and measured alone, as well as in the presence of phospholipids (data not shown), demonstrated that the reported method allows accurate protein quantification from lysis buffers. To determine if the reported method can be further applied to more complex biological samples, protein content in variously prepared breast cancer cell line (MCF-7) lysates was measured and compared to values derived from a BCA protein assay. Total protein content in the sample obtained using CytoBuster protein extraction reagent was estimated at 2.4 mg/mL (BCA assay) or 2.7 mg/mL (MIR-based method). Protein concentration in samples lysed with RIPA buffer was estimated at 3.4 mg/mL (BCA assay) or 4.6 mg/mL (MIR-based method). The results obtained using BCA assay were consistently lower (10% and 26%, resp.) than the MIR-based method. For the two methods, the differences in estimated protein concentration were most likely caused by a combination of the following factors: presence of various detergents (documented to influence the accuracy of BCA assay [43]) in lysis buffers, documented inaccuracy of BCA in the analysis of native proteins [44], an error associated with use of BSA as a standard in colorimetric assays [2], and the fact that accurate quantification of peptides by the BCA assay could not be achieved without additional sample manipulations that are incompatible with biological material [45]. The possibility that MIR-based quantification is inflated by signal from nucleic acids present in cell lysates was excluded because the amount of nucleic acids generally present in such mixtures [46] is significantly below the detection level of the reported method. The findings reported here are consistent with a previous report where the MIR-based method was used to quantify protein content in crude human skin carcinoma cell lysates [46] or to adjust protein concentration prior to mass spectrometry and NMR analysis [47, 48].

**Figure 5:**
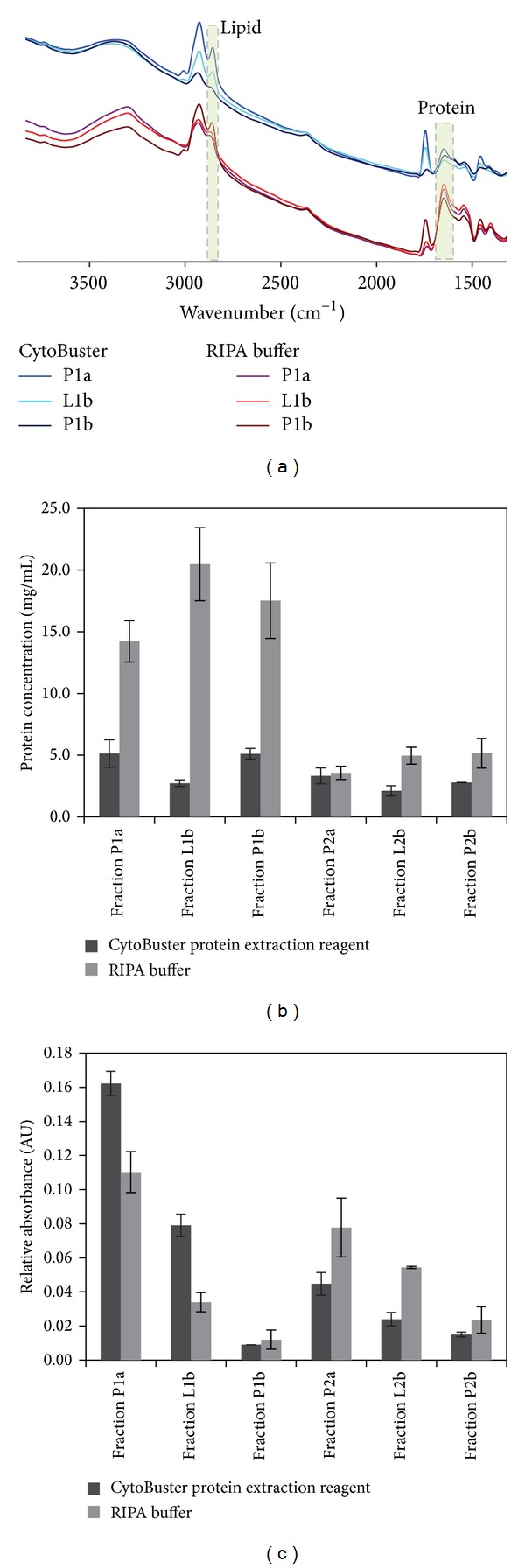
Application of the MIR-based univariate method for sample optimization in a short study of breast cancer biomarkers. The method was used to monitor the efficiency of fat removal and total protein liberation at each step of sample preparation from breast cancer tissue. Briefly, equal portions of breast cancer tissue were initially homogenized using either of two lysis buffers (RIPA or CytoBuster). Following homogenization, the samples were subjected to successive centrifugation steps. Resulting fractions were analyzed for protein and lipid content using the MIR-based method. (a) Overlay of raw MIR spectra collected for fraction 1 (P1a, L1b, and P1b, see below for definition) of breast cancer tissue lysed using RIPA buffer (violet, red, and brown) and CytoBuster protein extraction reagent (blue, cyan, and navy). In order to improve visualization, sets of curves representing each lysis buffer were manually separated. Areas utilized to quantify protein and analyze lipids are highlighted. (b) Total protein recovery across the centrifugal fractions. P1a and P2a represent bottom layer fraction from the first centrifugal spin. L1b and L2b represent top lipid containing fraction from the second spin. P1b and P2b show protein content in bottom layer fraction from second centrifugal spin. Total protein liberated using CytoBuster protein extraction reagent is shown in black while protein recovered using RIPA buffer is shown in grey. (c) Efficiency of gradual fat removal by consecutive spinning cycles. Fractions shown are the same as presented in B. Relative absorbance of lipid using CytoBuster protein extraction reagent is shown in black while protein recovered using RIPA buffer is shown in grey. Dual analysis of protein yield and fat content permits in-line optimization of the sample preparation process to meet the requirements of each downstream method of analysis.

The method described here was able to detect and examine protein and lipid content of all analyzed samples simultaneously and selectively (Figure 5(a)), in contrast with conventional analysis techniques such as UV spectroscopy. This dual functionality could enable the monitoring of changes in total lipid content and protein liberation yield, thereby simplifying and improving the analytical process. The efficacy of MIR-based analysis for use in downstream sample qualification was evaluated during a short study of breast cancer cell lysate fractionation and biomarker detection. Breast tissue was chosen because it is documented to have a high fat content (69.9 ± 22.9%) [49]. In the reported study, breast tissue lysates were prepared using RIPA buffer and CytoBuster protein extraction reagent. Following tissue homogenization, the efficiency of fat removal and total protein liberation during centrifugal extractions was monitored using the MIR-based method (see Supplementary Figure S2 for separation flow chart). The collected spectral data were used to determine protein recovery across the centrifugal fractions (Figure 5(b)) and to examine the efficiency of gradual fat removal from these samples (Figure 5(c)). Information delivered by MIR-based analysis was utilized in further sample manipulations facilitating faster and more consistent immunodetection of the investigated breast cancer biomarkers (study in progress).

## 4. Discussion

Fast and accurate quantification of proteins from complex aqueous biological samples such as plasma, cerebrospinal fluid, or cell/tissue lysates remains a challenge. Critical understanding of results from downstream applications is often dependent on proper preparation and accurate qualification of the applied samples. Classical methods for protein quantification, like UV and colorimetric assays, permit precise estimation of purified protein concentration but are less reliable when applied to the analysis of complex mixtures. While MIR is a promising technique allowing accurate quantification in complex samples, existing techniques for MIR-based protein quantification require regular time-consuming cleaning procedures (flow-through cells), larger sample volume (ATR cells), or expertise in method development (ATR of dried samples), thus precluding their suitability for fast analysis in a typical research laboratory. Although high-throughput MIR sampling and analysis techniques are already available on the market, they are more suited for larger pharmaceutical laboratories that require recurring analysis of repetitive sample sets and possess the requisite analytical skills for effective chemometrics-based method development. Currently available instrumentation is often too costly or requires certain IR expertise to be easily adapted for general protein quantification by non-IR savvy personnel in small-scale laboratories or academia. The method developed and described herein relies on a simple, cost-effective approach permitting accurate quantification results based on a univariate sample analysis technique that does not require advanced IR expertise.

The system relies on a membrane, transparent in most of the MIR region, which permits robust analysis of aqueous samples in a dried format. The reported method is also compatible with organic solvents commonly used in peptide and lipid research. To achieve accurate quantitative results, strict adherence to a consistent loading volume between samples and controls is required. While a general calibration curve can be employed, the method is more accurate when a specific calibration curve is prepared for each buffer to be utilized. Based on the experimental data, a general lower quantification range was set to 0.25 mg/mL; for some samples, a detection limit as low as 0.1 mg/mL can be achieved. Because the developed method focuses solely on small volumes and biologically relevant samples, the upper detection limit was not investigated thoroughly. However, from the narrow set of experimental results, such limits can exceed 100 mg/mL. Although some buffer salts might not be compatible with the reported technique, in PBS, the method allows development of a general linear calibration between 0.25 mg/mL and 5 mg/mL. Accuracy and precision of the reported method, within the linear range, are comparable with results obtained by amino acid analysis, providing researchers with a fast and cost-effective alternative to other protein quantitation tools available today.

Due to chemical and structural variation observed within lipids and detergents, MIR signal strengths display considerable variability; for this reason, a universal standard for all lipids and detergents could not be identified. Therefore, quantitation of lipids and detergents requires development of separate standard curves for each of the analyzed compounds. In addition, qualitative analysis of mixtures or unknown compounds can be performed using a “relative absorbance” mode where the method delivers information about strength of MIR signal without any comparison to a known standard.

In summary, the MIR-based method reported here enables simultaneous measurement of total protein recovery and monitoring of fat removal from lysed samples. This technique was also successfully applied to complex sample analysis during a small-scale investigation of surgical breast tissue processing and fractionation. MIR-based analysis facilitates more in-depth sample characterization and offers higher quality control over the sample preparation process. Given its unbiased biomolecular detection capabilities and amenability to liquid samples, many more applications are easily envisioned for this method.

## Supplementary Material

The supplementary material contains the evidence of the effects of buffer composition on the shape and distribution of the dried sample “coffee ring”. In addition a flow chart outlining the preparative workflow for protein biomarker fractionation from surgical breast cancer tissue is presented. Also, the analysis of the MIR signal produced by comparable concentrations of various lipids and detergents is provided.

## Figures and Tables

**Table 1:** Results of a review of the human protein database (Uniprot Release 2012_10). In total, 10 parameters were analyzed: length of the protein (Len), average molecular weight (MW), the average molecular weight per amino acid (Ave MW each AA), the number of glutamines (nQ), the number of asparagines (nN), the number of tryptophans (nW), the theoretical extinction coefficient (EC), both on a molar basis and on a mg/mL basis, the number of amide bonds, and the mass per amide bond (MW divided by amide bonds). For each parameter, the following statistical values were calculated based on all proteins in the database: average length, the standard deviation of the length (STDEV), maximum length (Max), minimum length (Min), and the coefficient of variation (CV; STDEV/average reported in percent).

	Len	MW (Da)	Ave MW each AA (Da)	nQ	nN	nW	EC (AU/(M cm))	EC (AU mL/(mg cm))	Amide bonds	Mass/Amide (Da)
Average	558	62164.2	111.5	27	20	7	59310	1.01	604	103.6
STDEV	603	66776.0	3.4	31	24	8	65792	0.51	653	3.4
Max.	34350	3816036.9	138.3	942	1111	466	3991820	5.26	36402	166.9
Min.	4	500.6	82.6	0	0	0	0	0.00	3	65.0
CV	108%	107%	3%	118%	121%	120%	111%	51%	108%	3%

Note that each of the calculated values was determined for each protein and then the statistics were calculated for each value across all proteins.
