# Supplementary figures and images for: Development of a Univariate Membrane-Based Mid-Infrared Method for Protein Quantitation and Total Lipid Content Analysis of Biological Samples
Source: J Anal Methods Chem. 2014 Oct 13;2014:657079. doi: 10.1155/2014/657079 (PMC4211209; doi:10.1155/2014/657079)

A

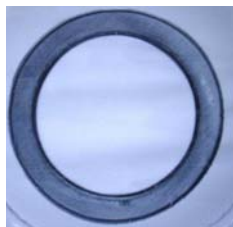

B

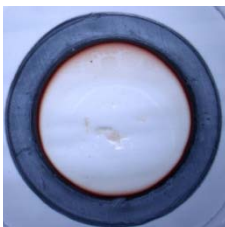

C

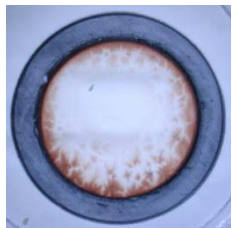

D

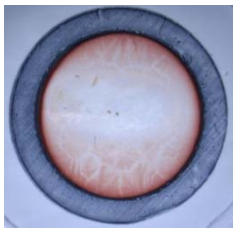

Supplementary Figure S1

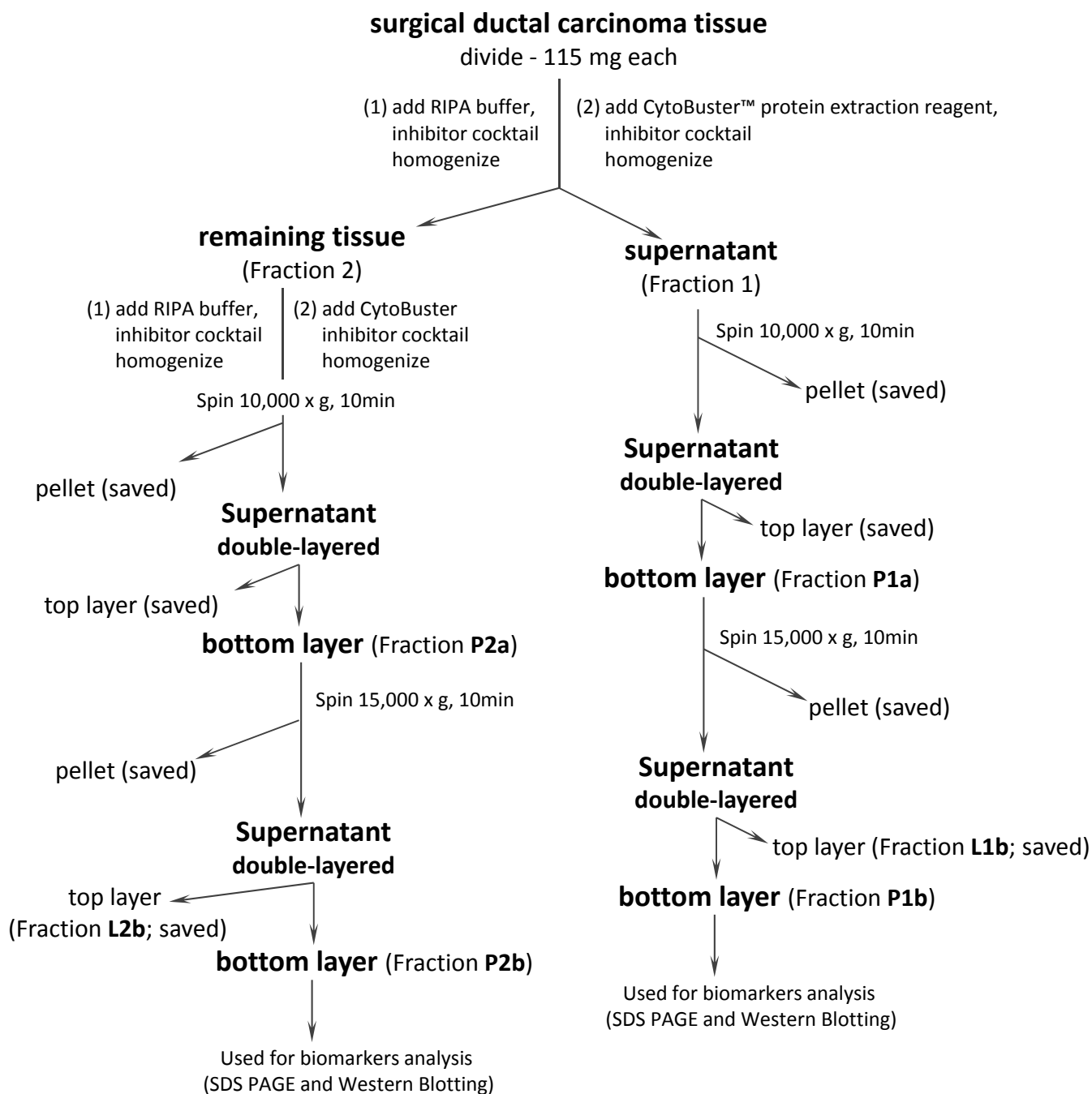

Supplementary Figure S2

A

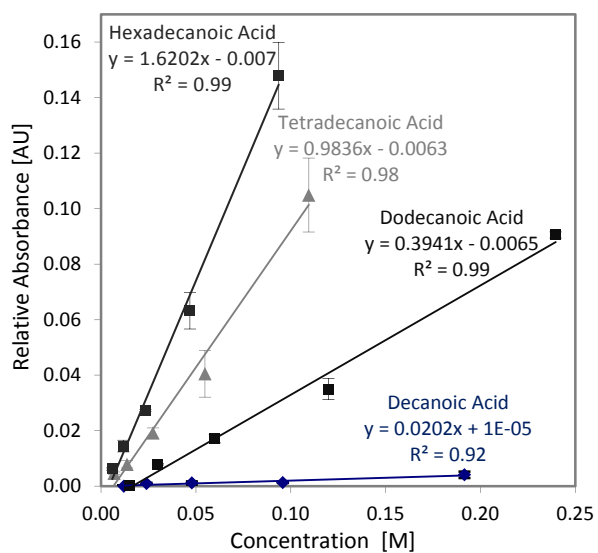

B

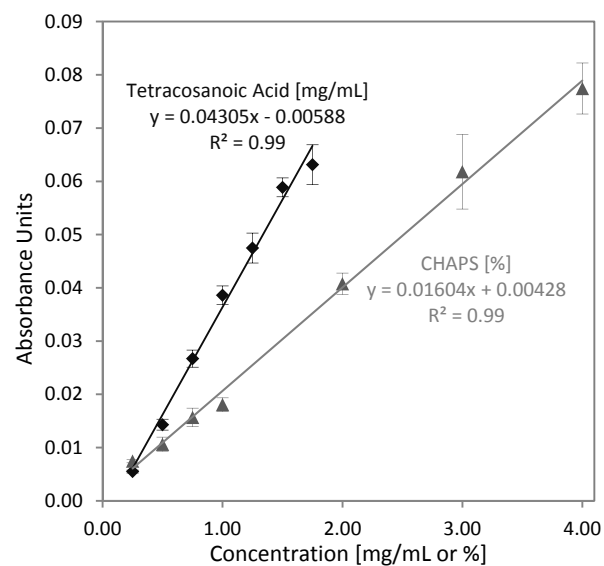

Supplementary Figure S3

Supplement: Supplementary file 1 — The supplementary material contains the evidence of the effects of buffer composition on the shape and distribution of the dried sample “coffee ring”. In addition a flow chart outlining the preparative workflow for protein biomarker fractionation from surgical breast cancer tissue is presented. Also, the analysis of the MIR signal produced by comparable concentrations of various lipids and detergents is provided. [file 657079.f1.pdf]
